# Asthma-like symptoms: is it always a pulmonary issue?

**DOI:** 10.1186/s40248-018-0136-5

**Published:** 2018-08-03

**Authors:** Davide Piloni, Claudio Tirelli, Rita Di Domenica, Valentina Conio, Amelia Grosso, Vanessa Ronzoni, Filippo Antonacci, Pasquale Totaro, Angelo G. Corsico

**Affiliations:** 1 Department of Medical Sciences and Infective Diseases, Unit of Respiratory Diseases, IRCCS Policlinico San Matteo Foundation, Pavia, Italy; 2 Thoracic surgery department, Columbia University, New York, USA; 3 Department of Internal Medicine, Section of Pneumology, University of Pavia, Pavia, Italy; 4 Cardiothoracic and Vascular Department, Unit of Cardiothoracic Surgery, IRCCS Policlinico San Matteo Foundation, Pavia, Italy

**Keywords:** Double aortic arch, Central airway, Spirometry, Persistent cough

## Abstract

**Background:**

Double aortic arch is a rare congenital and complete vascular ring around trachea and esophagus. It is usually diagnosed during infancy. The symptoms are generally related to respiratory and gastroesophageal tracts.

**Case presentation:**

A 20-year-old female patient was referred to our outpatient clinic for persistent dry cough. She had a history of an episode of inhalation of food bolus as an infant and recurrent bronchitis, anorexia and allergic bronchial asthma since the childhood. Since the beginning, an intrathoracic obstruction was suspected at pulmonary function tests. After 1 month of complete asthma treatment, the cough was unchanged and the spirometry confirmed the presence of an intrathoracic obstruction. Then, she underwent a chest CT with contrast medium, a contrast transthoracic echocardiography, a fiberbronchoscopy and an esophageal radiography with contrast medium. The final diagnosis was made and a double aortic arch was found.

**Conclusion:**

A careful observation of the flo*w*/*v*olume curve should always be guaranteed and the presence of congenital vascular anomalies should be suspected in case of difficult-to-treat asthma.

## Background

Double aortic arch (DAA) is a rare congenital aortic malformation resulting in an abnormal formation of a complete vascular ring around trachea and esophagus, normally diagnosed during infancy. The prevalence of the defect in the adult population is unknown. The DAA could be right-dominant (70%), left-dominant (25%) or balanced (5%). Symptoms, which are generally found during infancy, are caused by the aortic arch compression of the airways -with the presence of cough, dyspnea and wheezing- and/or of the digestive tract, with esophagus compression. Treatment is normally surgical for patients suffering from symptoms related to tracheal or esophageal compressions [1, 2].

## Case presentation

In October 2016, a 20-year-old female patient was referred to our outpatient clinic for persistent dry cough.

She reported that at the age of 16 months, because of the inhalation of food bolus, a bronchoscopy was unsuccessfully attempted and the episode resolved with a spontaneous expulsion of the foreign body. Thereafter, her medical history was characterized by recurrent bronchitis. At 11 y.o. she was diagnosed with allergic bronchial asthma (positive methacholine test and positive skin tests for both perennial and seasonal inhalation allergens such as dermatophagoides, cat, horse and pollens of grasses). The patient also reported a history of hypothyroidism and anorexia for which she had been admitted for a few months between 2013 and spring 2015 and fed by a naso-gastric tube.

**Fig. 1:**
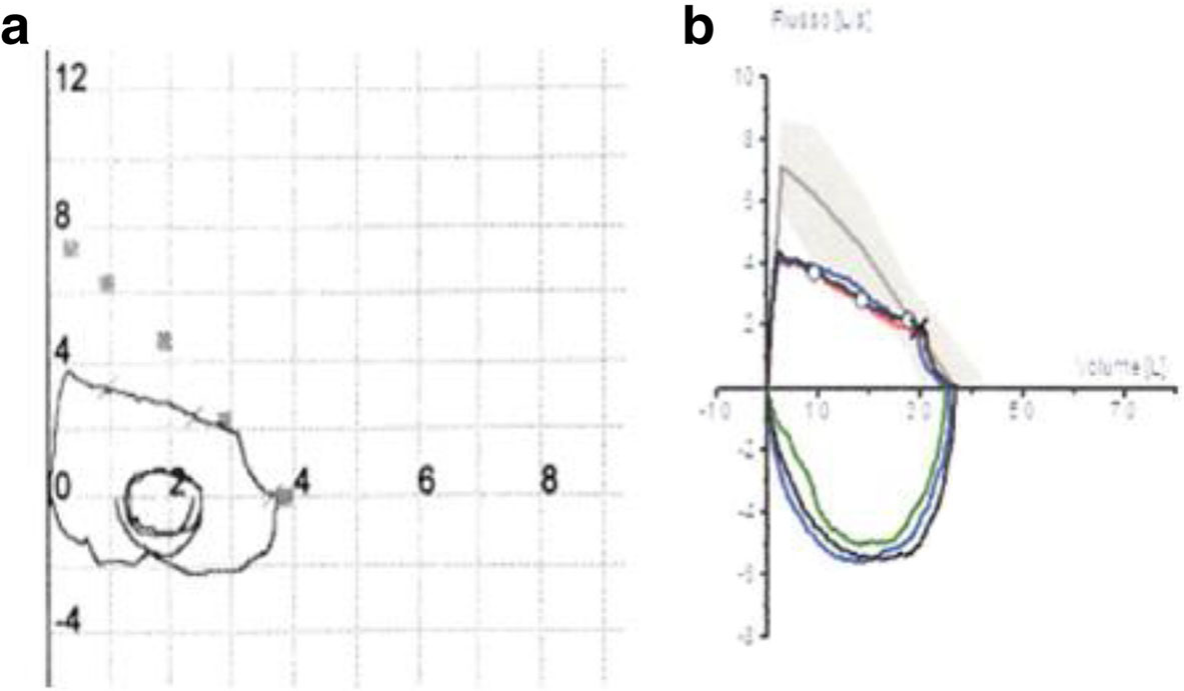
**a** flo*w*/*v*olume curve at the first pulmonary function test; **b** flow/volume curve after 1 month of therapy

At her first visit at our outpatients clinic, she complained of persistent irritating cough, which was accompanied by dysphonia in the last month. No dyspnea was reported. The pulmonary function tests showed: FEV_1_ of 2.88 L (85% of predicted), FVC 3.71 L (96% of predicted) with a FEV_1_/FVC ratio of 77% and an FEV_1_/PEF ratio > 8, as in cases of intrathoracic obstruction; furthermore, analysis of the flo*w*/*v*olume curve (Fig. 1a) showed a flow plateau of the expiratory curve with an armpit at low pulmonary volumes. Chest x-ray showed no pathological signs. A cycle of inhalation therapy with LABA and inhaled steroid was started. After 1 month of therapy, the patient was still complaining dry cough. A new spirometry showed findings similar to the previous one (Fig. 1b), thus confirming an intrathoracic obstruction.

**Fig. 2:**
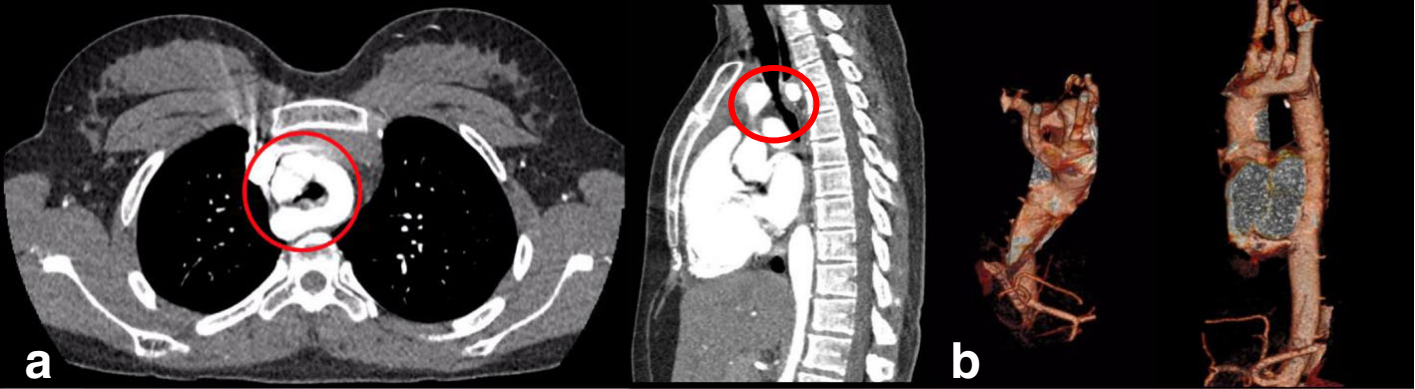
**a** CT scan with contrast medium highlighting the presence of a complete Double Aortic Arch (axial and sagittal reconstructions). Red circles indicate the 2 arches surrounding the trachea and esophagus. **b** Arterial phase CT: 3D reconstructions of the aorta, showing the DAA

The diagnosis was made after performing a chest CT with contrast medium. The CT demonstrated the presence of a complete double aortic arch (DAA) imprinting both the esophagus and the trachea, causing a greater narrowing in the expiratory phase (Fig. 2).

Then, the patient was referred to the cardiothoracic surgery unit for evaluation for a surgical DAA correction. During the hospitalization were performed: a contrast transthoracic echocardiography (showing a possible minimum pulmonary arterial-venous shunt), a fiberbronchoscopy (confirming *ab extrinseco *tracheal compression) and an esophageal radiography with contrast medium (showing a dilated upper esophagus followed by 30 mm of narrowed caliber). Given the important anatomical impairment and the poor quality of life due to the symptoms, a surgical correction of the DAA was performed, obtaining improvement of the symptoms.

## Conclusion

The peculiarity in this patient’s story is due to the late recognition of the DAA presence, around 20 years of age; even if the first signs could be retrospectively traced back to infancy for the recurrent bronchial infections and eating disorder. In this case, given the coexistence of bronchial asthma (as demonstrated by bronchial hyperreactivity during infancy), bronchodilators and inhaled steroids administration was misleading, resulting at first in a partial relief of respiratory symptoms. All these episodes during infancy could be retrospectively related to the anatomical anomaly [3]. The suspect of an *ab extrinseco* upper airways compression was based on two subsequent pulmonary function tests showing the persistence of a FEV_1_/PEF > 8 ratio with a saddleback at lower pulmonary volumes at the end of the expiratory plateau curve. Generally, typical flo*w*/*v*olume curve abnormalities are evident before the dynamic volumes pathologic reduction, but at the same time they become evident only when the central airway caliber lowers to a diameter of 8–10 mm [4, 5].

In conclusion, a careful observation of the flow/volume curve should always be guaranteed and the presence of congenital vascular anomalies should be suspected in case of difficult-to-treat asthma in absence of other possible causes that could explain treatment failure.

## Abbreviations

CT: Computed tomography
DAA: Double aortic arch
FEV_1_: Forced Expiratory volume in 1 s
FVC: Forced vital capacity
LABA: Long-acting beta-2 agonist
PEF: Peak expiratory flow
